# Integrated care for older people in Europe—latest trends and perceptions

**DOI:** 10.5334/ijic.831

**Published:** 2012-01-30

**Authors:** Kai Leichsenring

**Affiliations:** European Centre for Social Welfare Policy and Research, Berggasse 17, A-1090 Vienna, Austria

**Keywords:** integrated care, long-term care system, older people, practice examples

## Abstract

As a researcher and consultant I have coordinated local pilots and European research projects to analyse and improve long-term care for older people by better integrating health and social care systems. One of my conclusions from the wide range of initiatives that have been taken over the past two decades in Europe has been the need to treat long-term care as a system in its own right. Long-term care systems require a discernable identity; specific policies, structures, processes and pathways; and the leadership and resources that can underpin expectations, drive performance and achieve better outcomes for people that are living with (and working for those with) long-term care needs. Progress in developing LTC systems can be identified today in all European countries. Integrated care solutions at the interface between health and social care, and between formal and informal care, have appeared. These have been achieved partly by means of (slow) political reforms, partly as a response to market-oriented governance, and in many cases through pioneering community and civil society initiatives. It will depend on such initiatives, and their ability to convince both citizens and policy-makers, whether new societal approaches to long-term care are created that meet the demands of ageing societies.

## Introduction

As a researcher and policy-consultant I have focused much of my career on integrated care, starting at the beginning of the 1990s with regional pilot projects in Lower Austria to introduce local care coordination and counselling. Care coordinators had been installed in three catchment areas with about 15,000 inhabitants to provide information for people in need of care and their carers, to network with relevant stakeholders (from GPs and home care services to care homes and hospitals, but also pharmacies and the regional health insurance agency), and to improve awareness, cooperation and individual ‘care packages’. Case managers did not dispose of any additional budget for care as, in terms of resources, they should help families deal with the comprehensive long-term care allowance that had been introduced in 1993, shortly before the start of the pilot project.

Despite positive evaluations in terms of user satisfaction, networking and the start of new initiatives in local social care settings [1], these initiatives were crushed between political and sectoral cleavages. As the pilot project had been promoted by the Regional Councillor for Social Affairs from the Social Democratic Party, it was—almost as an automatic response—rejected and undermined by the Regional Councillor for Health from the Conservative Party. This conflict got even more complicated as most home care agencies in Austria are affiliated to the political parties. Concerning the professional cleavages, it was not easy to find GPs collaborating with the project. Although they complained about “too much time spent with older patients just visiting for a chat” they seemed to fear competition or loss of status, and some had communication problems due to the professional background of the case managers (social workers). Similar challenges had to be faced with hospital staff.

As a result of my experiences in Lower Austria and comparative studies about payments for care and other innovations in social and health care in Europe [2, 3]. I wanted to learn more about integrating social and health care, and how this challenge was tackled in other countries. An EU project “Providing Integrated Health and Social Care for Older People” (PROCARE) which I coordinated and carried out with colleagues from nine European countries from 2002 to 2005 [4, 5] showed that “national health and social care systems remain—at best—loosely coupled systems that are facing increasing difficulties, given the current challenges, in particular in long-term care for older persons: increasing marketization, lack of managerial knowledge (co-operation, co-ordination), shortage of care workers and a general trend towards down-sizing of social care services continue to hamper the first tentative pathways towards integrated care systems” [6]. However, based on the findings of this project, we went on to search for elements of ‘emerging long-term care systems’ by modelling and illustrating a systematic framework for long-term care with colleagues from 13 European countries [7]. INTERLINKS which was funded under the 7th Framework Programme of the European Commission has been another important step to improve the knowledge base for prevention and rehabilitation, informal care, quality of services as well as governance and financing of long-term care.

This perspectives paper aims to reflect upon my experience in researching long-term care, the rationale for creating integrated long-term care systems, and to showcase some innovations needing further research and development.

## Towards an integrated system of long-term care for older people?

Systems theory protagonists have dealt with health and long-term care only marginally. Yet they underline that health systems’ success is based on its clearly defined function to cure patients from illness [8]. Health systems reveal, however, difficulties in coping with chronic diseases, disabilities and situations that necessitate long-term care and the communication with a wide range of stakeholders beyond their boundaries [9]. This is why social innovation in terms of making a functional differentiation towards integrated LTC systems with their own identity is an appropriate approach since it calls for specific structures, processes and resources that connect to the ‘lifeworld’, as Habermas [10] coined it, of older people in need of care, their families and informal carers.

Based on more than 30 national reports, European overviews and working papers the INTERLINKS consortium constructed a framework for an ‘ideal-type’ long-term care system (see the INTERLINKS project website at http://interlinks.euro.centre.org/ which shows how all the different types of care for older people need to be identified, co-ordinated and managed to improve person-centred care). Altogether, the ‘ideal-type’ INTERLINKS framework for long-term care brings together 135 key issues under six ‘framework themes’ that have to be considered to analyse and improve existing systems at the interfaces between health and social care. Apart from the need to give an ‘identity’ to long-term care (as argued above) the themes comprise: policy and governance, organisational structures, management and leadership, processes and pathways as well as means and resources.

To give an example of the depth and complexity to the long-term care system components identified, the theme ‘organisational structures’ has a number of sub-themes including ‘formal care in the home and community’ within which the following key-issues were defined:

- access points (referral, counselling, one-stop-shops)
- flexible and adaptable services to suit individual needs and individual lifestyle
- multi-professional teams (e.g., preventive/rehabilitative measures)
- structures that facilitate coordination and cooperation with other formal and/or informal care (including mobility and transport)
- structures that facilitate communication, planning and care delivery with informal carers
- practitioners in independent practice as gate keepers and/or personal case and care managers
- diversity-friendliness: recognition of the specific care needs of hard-to-reach groups, especially their specific needs for information, coordination and support to access available services and benefits.

What may sound here to be a highly abstract exercise for the research project has actually been supplemented by a focus on the practical aspects of long-term care from the viewpoint of users, carers and all other stakeholders involved in care delivery. This is underpinned by the description and analysis of almost 100 innovative practice examples that illustrate how these key issues can be addressed in a meaningful way by focusing on users and the need for coordination and integration.

The framework and the practice examples have been validated by members of a Sounding Board, representing European stakeholder organisations in ageing and long-term care, and by the members of National Expert Panels in 13 participating countries.

The INTERLINKS framework for long-term care wants to inspire policy-makers and practitioners to work towards integrated long-term care systems by learning from validated practice examples with a focus on prevention and rehabilitation, the development of quality management and support for informal carers. The framework helps to find innovative ways to provide integrated care and to learn from other countries—in other words, to meet an objective that has been formulated in countless research and policy-papers in the past. Having been involved with many long-term care developments over the past 20 years, my experience is that it usually takes at least 10–15 years for pioneering approaches to get acknowledged in one country and often even longer to cross borders. In addition, concepts such as integrated care have many meanings even within the health care system [11]. Tools and methods for a quicker information exchange, cross-border evaluations and adaptations, including the development of a common understanding, are therefore urgently needed. EU research projects such as INTERLINKS may partly contribute, but even within the group of interdisciplinary experts participating it took almost the entire project period to agree on terms and approaches. National idiosyncrasies often prevail on shared findings even in research, not to speak of national policies and governance. This slightly negative stance, however, should not prevent us from further efforts to bring about change at the interfaces between health and long-term care.

## Practice examples

The INTERLINKS project has identified a wide range of practice-based examples that work towards better linkage, coordination and integration of long-term care—at various levels and addressing different sub-themes and key-issues that have to be developed in order to bring about long-term care systems. For instance, on the systems level, governance mechanisms that make appropriate government levels accountable for providing an appropriate infrastructure (e.g., Sweden, Denmark, partly England) or that allow funding for individuals with long-term care needs (e.g., Austria, France, Germany) as well as, on a local level, for informal, including migrant carers (e.g., first steps in Italy, Spain).

Also, mechanisms to better link prevention and rehabilitation to long-term care, including new job profiles such as case management and ‘Everyday Assistants’ (Germany) or community nurses promoting person-centred care (e.g., Netherlands) are an important ingredient to generate new perspectives towards integrated long-term care. Among the wide range of innovative approaches—always recalling that national differences for innovation have to be considered before transferring particular ways of working from one country to another—one example for flexible and adaptable services to suit individual needs and individual lifestyle should be specially highlighted: ‘Care in the neighbourhood’ (‘Buurtzorg’) is an example from the Netherlands that shows particularly well that there are opportunities for a quick roll-out of new ideas if they prove their quality to be client-oriented and cost-efficient.

The Buurtzorg model [12, 13] was designed by experienced district nurses in 2006 with the objective to provide integrated home care, i.e., with connections to social services, general practitioners, and other providers, for all persons who need care at home. Care is delivered by small self-managing teams with a maximum of 12 professionals. To keep organisational costs as low as possible, ICT is used for the organisation of care with a small but efficient centralised back-office. The Buurtzorg method has six sequential components, which are delivered as a coherent package and cannot be delivered separately. The package includes assessment, mapping and involving the network of informal care as well as formal carers, care delivery, support of the client in his/her social roles and the promotion of self-care and independence. The model was introduced on the strictly regulated quasi-market of Dutch home care and had to compete with usual providers for clients and contracts. By mid-2010, teams were active in 250 locations, with a total number of staff in these teams of 2600 (amongst them 1500 qualified district nurses) who serve about 30,000 clients annually. The growth rate of Buurtzorg has continued since with about 70 staff members in 5–10 teams per month. The centralised back-office consists of about 30 professionals. Today, Buurtzorg ranks number 1 amongst all home care organisations in user satisfaction according to results of the mandatory national quality of care assessment. In 2011, the organisation has been awarded a prize as the best employer of the Netherlands in organisations with now more than 4000 employees. A significant result is the impressive decrease of costs that seem to be less than half than those for usual home care [14]. Buurtzorg may be setting a new standard for home care in the Netherlands. Its main strength is to successfully bridge gaps in local level home care.

## Conclusions

Further reforms will show whether long-term care issues continue to be subsumed to the health care system or if their specific features will result in long-term care gaining its own identity focusing on user involvement, quality of life and always considering respective links to neighbouring systems such as social and health care, housing and employment. Integrating long-term care to meet the general challenges of ageing societies will remain an exciting area for further organisational development, training and research. It will therefore also be an important topic during the 2012 “European Year for Active Ageing and Solidarity Between Generations” [15]—this will also be an opportunity for all of us who have worked for better integrating health and social care towards a long-term care system with the people concerned.
